# Concomitant Production of Lipids and Carotenoids in *Rhodosporidium toruloides* under Osmotic Stress Using Response Surface Methodology

**DOI:** 10.3389/fmicb.2016.01686

**Published:** 2016-10-25

**Authors:** Gunjan Singh, Arshad Jawed, Debarati Paul, Kalyan K. Bandyopadhyay, Abha Kumari, Shafiul Haque

**Affiliations:** ^1^Amity Institute of Biotechnology, Amity UniversityNoida, India; ^2^GE HealthcareGurgaon, India; ^3^Department of Biosciences, Jamia Millia IslamiaNew Delhi, India

**Keywords:** biofuels, *Rhodosporidium toruloides*, osmotic stress, RSM

## Abstract

As a replacement to existing fossil fuels, biofuels, have proven their worth; however, their widespread use is limited due to inconsistent yields, higher costs and poor productivity. An oleaginous yeast, *Rhodosporidium toruloides* has been reported to accumulate substantial amounts of lipids (that can be converted to biofuels) and therefore, it was selected for study and optimization. Apart from lipids, *R. toruloides* is also reported to produce carotene that can be used as a therapeutic agent. In this study, the culture medium was statistically modeled and optimized for concomitant production of lipids and carotenoids and for improving and maximizing the productivity of lipids as well as carotenes. The two metabolites were expressed differentially in the growth cycle of the organism. Culture medium components were simultaneously varied at five different levels using statistical modeling employing response surface methodology (RSM). Osmotic stress was introduced in order to simulate saline conditions and optimize the carotenoid as well as lipid production process, to be used in conditions with high salt contents. We observed a 10% (w/v) increase in carotenoid production in initial experiments under osmotic stress due to high salt concentration, while the increase in lipid synthesis was not pronounced. In this study, we demonstrate 36.2% (w/v) lipid production and 27.2% (w/v) carotenoid production, under osmotic stress with high salt concentrations, for the first time.

## Introduction

Hydrocarbons derived from biological sources hold significant promise as potential alternative for petroleum-based fossil fuels. Biodiesel comprises of fatty acid alkyl/methyl esters (FAAEs/FAMEs) produced via alkali- and/ or acid- catalyzed transesterification of lipids using ethyl alcohol/ methanol. Fatty acids, in the range of C_10_–C_18_ are excellent substrates for biodiesel production (Najafi et al., 2007). These substrates assure lower sulfur content, reduced carbon mono-oxide, and hydrocarbon emissions as compared to fossil fuels. However, commonly produced lipids in the biological system (include mono, tri-glycerides, phospholipids, and free fatty acids) are not a viable option for large-scale production as these suffer severely low yields.

A plethora of reports are available on the synthesis of first generation biofuels, derived mainly from plant oils. However, as growth and maturation of plants are directed by environmental factors, yields normally vary (Takeshita, 2011). This limits the use of plants for large-scale production of biofuels. Alternatively, microbial lipid production has short cultivation and production cycle, remains unaffected by environmental factors, offers simple containment and disposal, easy to culture and scale-up without the limitations posed by the first and second generation biofuels (Azocar et al., 2010; Rossi et al., 2011). Among heterotrophic micro-organisms oleaginous yeasts have been reported to be decent producers of lipids, with a higher ratio of triacylglycerols (TAGs). Oleaginous yeasts are capable of synthesizing and accumulating high amounts of neutral lipids up to 70% of biomass (Czabany et al., 2007). A few species of oleaginous yeasts have been identified and studied within the genera *Yarrowia, Candida, Rhodotorula, Rhodosporidium, Cryptococcus*, and *Lypomyces* (Amaretti et al., 2010; Ageitos et al., 2011; Rossi et al., 2011; Almanza et al., 2014). *Rhodosporidium toruloides* has conveniently been grown in bioreactors on various media based on waste-water, waste juices, etc., for the production of microbial lipids. *Rhodosporidium glutinis* (teleomorph for *R. toruloides*) accumulates lipids up to ~50% of its total cell weight, during the log phase and amasses significant amounts of carotenoids during the stationary phase of growth. Carotenoids are important as these can be used as vitamin-A precursors, natural dyes, antioxidants, and possible tumor-inhibiting activity, proving their potential for the pharmaceutical, chemical, food, and feed industries (Baker and Guenther, 2004; Frengova and Beshkova, 2009). Epidemiological evidences and experimental data suggest that carotenoids in food inhibit the onset of many diseases, such as arteriosclerosis, cataracts, multiple sclerosis, and cancer (Forman et al., 2004; Frengova and Beshkova, 2009). The synthesis of different natural commercially important carotenoids (β-carotene, torulene, torularhodin, and astaxanthin) by *R. toruloides* has led to the consideration of this organism as a potent source of carotenoids having medical and commercial interest. Stringent culture conditions are required by oleaginous yeasts to induce lipogenesis, with C/N ratio skewed excessively toward carbon, creating nitrogen limitation in the culture medium (Ratledge and Wynn, 2002). Carotenoid accumulation in most yeast starts in the late logarithmic phase and continues till the conclusion of stationary phase (Goodwin, 1972). The requirement of carbon source for carotenoid production is crucial for carotenoid biosynthesis during the stationary phase (Frengova and Beshkova, 2009).

In this study, we attempted to maximize biomass, concomitant production of lipids, and carotene by statistical modeling and optimizing the culture media. The effect of salinity and its interaction with other media components and on cell growth and lipid/carotene production using advanced statistical modeling methods, i.e., response surface model (RSM) was attempted. High salinity damages the cell wall of yeast cells due to high osmolarity, making it a critical parameter to be optimized accurately. Osmotic stress has been shown to affect cellular metabolism at various levels, initiate translation inhibition, and sometimes represses polysomal association of mRNA, hence affecting the transcript levels in the cells (Melamed et al., 2008). The culture medium is a complex formulation and the components are expected to interact with each other in an intricate manner. The microbial cells too, behave in a complicated fashion, switching their preference for one component over the others with changes in culture conditions. Presence of complex nutrients along with other media components facilitates the culture with ready-made nutrients and help accelerate the cell growth and metabolite production in a synergistic manner. As the cells do not need to manufacture many nutrients themselves, their adaptation and cell growth proceeds much quickly and rapidly (Manowattana et al., 2015). RSM was applied to study the interaction of the media components on cell growth and lipid/carotene production. Elevated intracellular ionic concentrations are often toxic for cells, however, in a *Fusarium* sp. isolated from saline soil, rearrangement of membrane lipids and accumulation of arabitol helps it to survive salt stress (Smolyanyuk et al., 2013). We expected changes in survival patterns and in growth profile, and lipid accumulation due to salt stress induced by the culture medium. Several reports are available for optimization of growth and lipid and carotenoid production (Bhosale and Gadre, 2001, 2002; Tinoi et al., 2005) from various strains of *Rhodosporidium* sp., however, the interplay of salinity and glucose and corresponding C/N ratio(s) has never been studied in the context to concomitant lipid and carotenoid production. In this study, we have also compared our strain to *R. glutinis* on several occasions as they are teleomorphs (Oloke, 2006).

In laboratories world-over, for optimization of media and process variables, one factor (process variable) is changed at a time (OFAT), while all other process variables are maintained at a specific/constant level either arbitrarily or by sequential OFAT experiments performed earlier (Cocaign-Bousquet et al., 1995). OFAT experiments are easy and less laborious but these suffer from being inaccurate, time consuming, need large number of runs, and do not account for the interaction(s) in between the media components and process parameters involved in the study. Statistical optimizations such as RSM are studies where participating parameters are varied simultaneously for optimization experiments (Greasham and Inamine, 1986; Greasham and Herber, 1997; Zhang et al., 2000; Margarita et al., 2005). RSM can collectively optimize all the affecting parameters to eliminate the limitations with single-factor optimization process (Gough et al., 1996; Dubey et al., 2011; Kanmani et al., 2013). We therefore, attempted to decipher and model glucose and NaCl content of the growth medium, using *R. toruloides.* The target was to maximize lipid and carotene accumulation by using central composite design (CCD) from the RSM group. RSM uses statistical tools such as multi-level factorial designs and multiple regression analysis to study the interaction between the crucial variables by evaluating process parameters (Weuster-Botz, 2000). Keeping all the facts in view, the present study aims to statistically model and elucidate interactions of media components and develop a robust and cost-effective method in saline environments for growth of *R. toruloides*, extraction of lipids and carotenoids for maximum productivity and yield.

## Experimental section

### Microorganism

*Rhodosporidium toruloides* (ATCC 204091), an oleaginous oxidative red yeast was maintained at 4°C on agar slant supplemented with 20 g/L glucose, 10 g/L yeast extract, 20 g/L peptone, and 20 g/L agar.

### Cell growth and culture conditions for *R. toruloides*

To cultivate *R. toruloides* the medium used was minimal media (MM; glucose 5 g/l; Na_2_HPO_4_ 6 g/l; NaCl 5 g/l; KH_2_PO_4_ 3 g/l; NH_4_Cl 2 g/l; MgSO_4_ 0.1 g/l; yeast extract 2 g/l) solidified with 1.5% agar. The culture was maintained at RT (~25–30°C) and shaking speed was 120 rpm. Growth was measured using optical density at 600 nm (Shimadzu UV-1650) and by quantitating biomass. A 48 h old culture at 10% (v/v) was used as inoculum for all studies. To obtain biomass, cells were harvested by centrifugation, washed with de-ionized water and dried at 85 ± 3°C and expressed as g/L. The effect of NaCl supplementation was studied with 1, 5, and 10% NaCl (w/v); the control media was devoid of NaCl. The residual amount of reducing sugar was estimated using 3,5 dinitrosalysilic acid (DNSA) method (Miller, 1959) in the fermentation media.

### Sudan black staining and microscopy

Cells obtained at various stages were stained with Sudan Black B as described by Chetana et al. (2015) for the detection and quantification of accumulated lipids. The stained cells were observed microscopically at 100X magnification.

### Determination of lipid content

Lipids were extracted by the method of Bligh and Dyer (1959). The cells (0.1–1.0 g) were harvested by centrifugation, washed/ dried to obtain constant weight; weight of the dry pellet was recorded (W). The dry pellet was resuspended in 3.75 ml of chloroform/methanol (2:1) vortexed for 15 min, added 1.25 ml of chloroform, vortexed again for 1 min, and lastly 1.25 ml of 1M NaCl was added. The mixture was centrifuged at 3000 rpm for 15 min to separate the aqueous and organic phase. The organic phase was transferred to weighed vial (W_1_) and dried till organic phase is evaporated. The weight of the vial was again recorded (W_2_). Lipid content was calculated as:

$$\begin{array}{l} \left({\text{W}}_{2}-{\text{W}}_{1}\right)/\text{ W} \end{array}$$

and expressed as % dry cell weight (W = weight of pelleted cells).

### Carotenoid determination and extraction

A modified protocol was followed for carotenoid extraction (Latha et al., 2005). Cells collected from about 1 ml culture were washed, resuspended in 2 ml of DMSO and incubated at 50°C for 1 h. The cells were again centrifuged at 3000 rpm for 10 min to collect the supernatant. The process was repeated until the cells became colorless. The carotenoid content was calculated and expressed as % dry cell weight using the formula:

(Weight of carotenoids extracted/Weight of pelleted cells).

### Selection and optimization of media components

Process optimization was carried out initially by traditional one-factor at a time approach by varying different media components. Cell growth, intra-cellular lipid accumulation, and carotene production was estimated discretely at different points of time, as defined by the experimental protocol.

### Statistical optimization of process parameters

Based on the results obtained from OFAT experiments, RSM experiments were planned with the selected process parameters. A three factor CCD was selected from the RSM. For setting up the RSM design matrix glucose (g/L), NaCl (g/L), and culture time (h) were chosen. The ranges selected were as shown in the (Table 1). The low and high limits define the boundaries of design space. Selected parameters were varied at five different levels (coded as −2, −1, 0, +1, +2). The variations were done simultaneously to generate a combination of the factors to be run as a single experiment as indicated in Table 2. A total of 18 experiments were generated. All combinations were run in two batches in triplicates. The amount of growth, lipid, and carotene produced was averaged, and fed in the combination matrix of the generated design against each run as shown in Table 2. The CCD model developed was used to navigate the design space forming a cube with different combinations of the three experimental parameters. The design includes center points, replicates, points along the median, edge and axial positions. Multiple regression analysis on the data obtained was performed by using design expert (STATEASE Inc., USA). The responses followed a quadratic model that can be represented by the following equation:

$$\begin{array}{l} Y=a_{0}+a_{1}*A+a_{2}*B+a_{3}*C+a_{4}*AB+a_{5}*AC\\ \;+\;a_{6}*BC+a_{7}*A^{2}+a_{8}*B^{2}+a_{9}*C^{2}. \end{array}$$

**Table 1:** **The experimental design parameters and constrains of design space for participating factors**.

**Factor**	**Name**	**Units**	**Type**	**Low actual**	**High actual**	**Low coded**	**High coded**	**Mean**	**Std. dev**.
A	Glucose	g/L	Numeric	5	10	−1	1	7.5	2.17
B	NaCl	g/L	Numeric	8	18	−1	1	13	4.35
C	Culture Time	h	Numeric	16	26	−1	1	21	4.35

**Table 2:** **Design of experiments with actual and predicted values of obtained growth, carotene, and lipid in each run**.

**Sr. No**.	**Experimental parameters**			**Actual values**			**Predicted values**		
	**Glucose (g/L)**	**NaCl (g/L)**	**Culture time (h)**	**Growth (g/L)**	**Carotene (%, w/w)**	**Lipid (%, w/w)**	**Growth (g/L)**	**Carotene (%, w/w)**	**Lipid (%, w/w)**
1	10.00	18.00	26.00	46.700	36.904	26.490	46.892	36.481	26.177
2	7.50	13.00	12.59	31.230	5.000	19.300	30.655	2.128	19.292
3	7.50	13.00	29.41	44.700	20.320	25.760	44.423	22.429	25.945
4	10.00	18.00	16.00	39.400	17.320	26.490	41.067	19.409	26.421
5	5.00	8.00	26.00	25.330	13.660	25.450	25.558	10.950	25.394
6	7.50	13.00	21.00	30.400	26.664	24.396	30.437	26.696	24.389
7	10.00	8.00	26.00	43.700	25.790	23.970	45.214	25.026	23.721
8	5.00	18.00	26.00	32.010	14.509	21.110	31.031	15.295	21.315
9	7.50	13.00	21.00	30.400	26.664	24.396	30.437	26.696	24.389
10	5.00	8.00	16.00	15.890	1.758	17.050	15.008	3.879	17.238
11	3.30	13.00	21.00	15.560	8.210	18.680	16.594	8.267	18.346
12	10.00	8.00	16.00	39.100	7.043	21.740	39.389	7.955	21.409
13	7.50	4.59	21.00	25.120	11.230	21.540	24.727	11.750	21.746
14	7.50	13.00	21.00	30.400	26.664	24.396	30.437	26.696	24.389
15	7.50	13.00	21.00	30.400	26.664	24.396	30.437	26.696	24.389
16	5.00	18.00	16.00	20.100	8.082	15.590	20.482	8.224	15.714
17	11.70	13.00	21.00	52.320	30.329	25.430	50.433	29.509	25.942
18	7.50	21.41	21.00	31.200	26.320	22.560	30.741	25.036	22.531

In this equation, Y is the corresponding product yield, e.g., growth, carotene, and lipid. A, B, and C are independent variables as defined previously and interaction effects between individual variables are shown by the interaction terms as AB, AC, and BC. The regression model is rotatable along the center and at the axial points, and can be used to calculate and understand the interaction in between the multiple independent variables, varied within the specified design space. The values of responses (mainly recoveries) obtained by experiments as indicated by the response surface model design, were statistically assessed using Analysis Of Variance (ANOVA) and significance values of the model were determined by using Fischer's test value (*F*-value; Table 3). The regression fit of the data obtained was appraised using regression coefficient (*R*^2^). The responses were represented by contour plots generated by inserting the response data obtained as per the model made by Design-Expert™. These contour/3D surface plots (**Figure 4**) are crucial for navigation of the experimental design space and for obtaining a proper composition of solvents, at their individual levels, that would ultimately result in higher recoveries of lipids and carotenoids from cell free fermentation triplicate.

**Table 3:** **Modeling parameters and ANOVA for growth, carotene and lipid synthesis**.

**Sl**.	**Parameter**	**Growth**	**Carotene**	**lipid**
1	*F*-value of RSM model	160.77	57.71	195.09
2	*p*-value of RSM model	< 0.0001	< 0.0001	< 0.0001
3	df	8	8	9
4	Lack of Fit	Not Significant	Not Significant	Not Significant
5	Pure error	0.00	0.00	0.00
6	Std. dev.	1.18	1.94	0.320
7	Mean	32.44	18.51	22.71
8	*R*^2^ (Coefficient of determination)	0.993	0.980	0.995
9	Adjusted *R*^2^	0.986	0.963	0.990
10	Predicted *R*^2^	0.961	0.893	0.965
11	Adequate precision	42.45	25.092	44.810

## Results

### Growth and residual glucose profile

**Figure 1:**
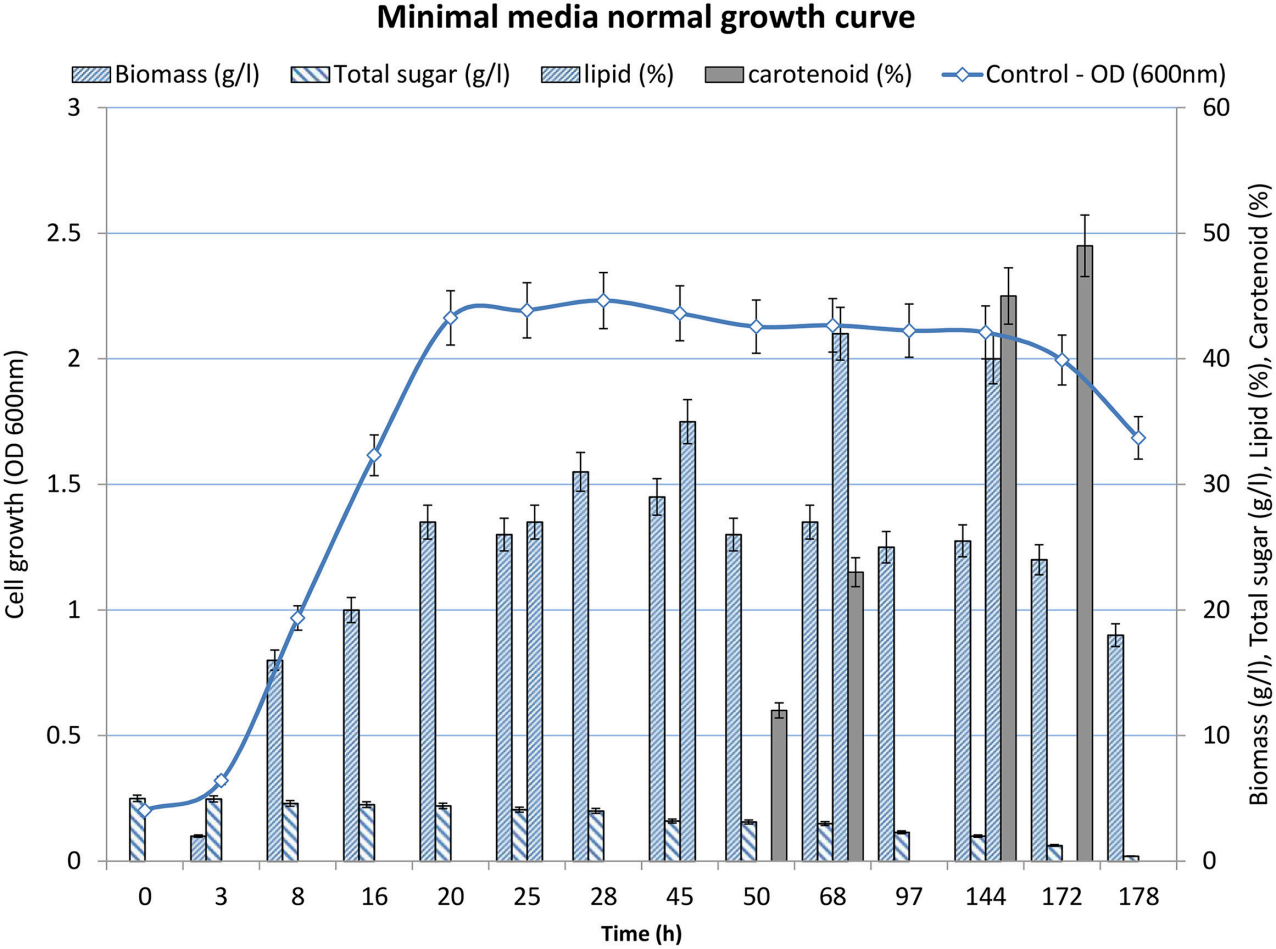
**Growth profile of *R. toruloides* showing increase in OD_(600*nm*)_ over time with depletion of glucose**.

Bioprocesses development for lipid production was designed around the ability of *R. toruloides* to grow and accumulate large amounts of lipids and carotene in the initial growth phase and late log to stationary phase of fermentation, respectively. When cultivated in minimal medium (Figure 1), the lag phase continued for 3 h and then the cells entered in to log phase that continued till 20 h of growth. A steady dip in the growth rate marked the beginning of the stationary phase (Figure 1). After 172 h, the cells moved in decline phase as the biomass decreased with elapsed time. Glucose (5 g/L) was provided in the medium as the carbon source to the culture. The residual level of sugar in the culture broth was measured at regular intervals to determine the substrate utilization rate and specific growth rate of the organism. The glucose utilization increased after the log phase constantly until 20 h at the rate of 0.032 gL^−1^/h). Glucose utilization continued at a slower rate during the stationary phase (0.017 gL^−1^/ h). The stationary phase ended at 140 h and marked the beginning of decline phase. Upon the onset of decline phase, residual sugar remained in the broth remained constant at the level of 1–2 g/L. Therefore, the culture was harvested at 140 h.

### Lipid production and carotene production profile

**Figure 2:**
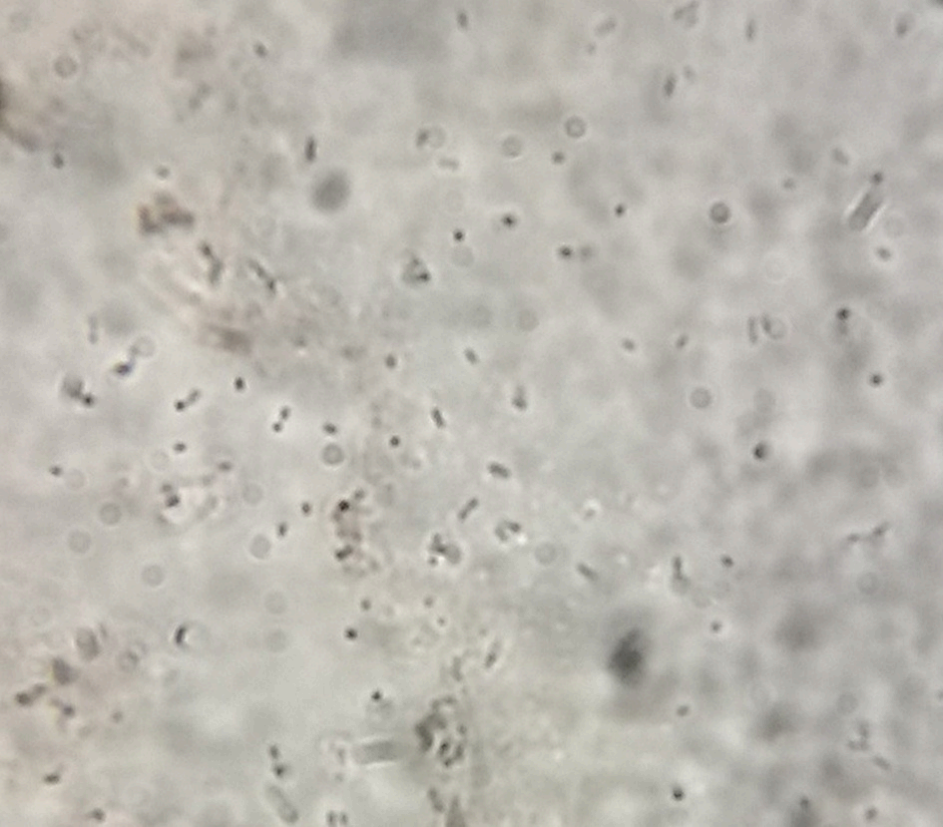
**Oil globules stained with Sudan B Black in *R. toruloides* cells**.

Samples (2 ml each) were withdrawn every 6 h to collect the cells for lipid/ carotene extraction and quantification. Until 24 h of growth, there was no significant accumulation of lipids as determined by staining the cells with Sudan dye. However, beginning at 25 h and continuing until ~70 h, lipid accumulation increased significantly as shown by increase in Sudan Black B stained lipid globules (Figure 2). Maximum lipid accumulation mounted to 42% (w/w) of total biomass after 68 h of incubation. Subsequently, at 48 h of growth, *R. toruloides* cells started to accumulate carotenoids, as visible by the development of pink color of the culture. Approximately 49% (w/w) carotenoids accumulated after 50–172 h of cultivation.

### Optimization of C/N ratio for maximizing biomass production

Variation of C/N ratios has been reported to affect the lipid accumulation of algae, yeasts, and other microbes. *R. glutinis* accumulated more than 50% lipids when C/N ratios were standardized in the medium from 10:1 to 70:1 (Braunwald et al., 2013). Lower glucose content in the medium matched with decrease in biomass, resulting in reduced lipid, and carotenoid accumulation (Supplementary Figure 1). On the other hand, glucose concentration < 5 g/l led to reduced growth, resulting in decreased production of biomass and lipids. Cell growth approaches stationary phase after 20 h of incubation whereas carotenoid production began after 50 h of growth. This indicates that variations in C/N ratios was not sufficient alone to induce concomitant production of higher amounts of lipid and carotenoids. Therefore, osmotic stress was attempted by addition of NaCl, to check if the growth profile was altered and the production of lipids and carotenoids changed to concomitant production. Keeping the glucose concentration constant at its optima, the culture media was supplemented with different concentrations of ammonium sulfate (Braunwald et al., 2013) or NaCl separately. As ammonium sulfate showed negligible effect on the production of lipids or carotenoids compared to NaCl, further studies were carried out with NaCl supplementation and considered as a crucial modeling parameter for RSM studies (data not shown).

### Optimization of lipid and carotenoid production OFAT

**Figure 3:**
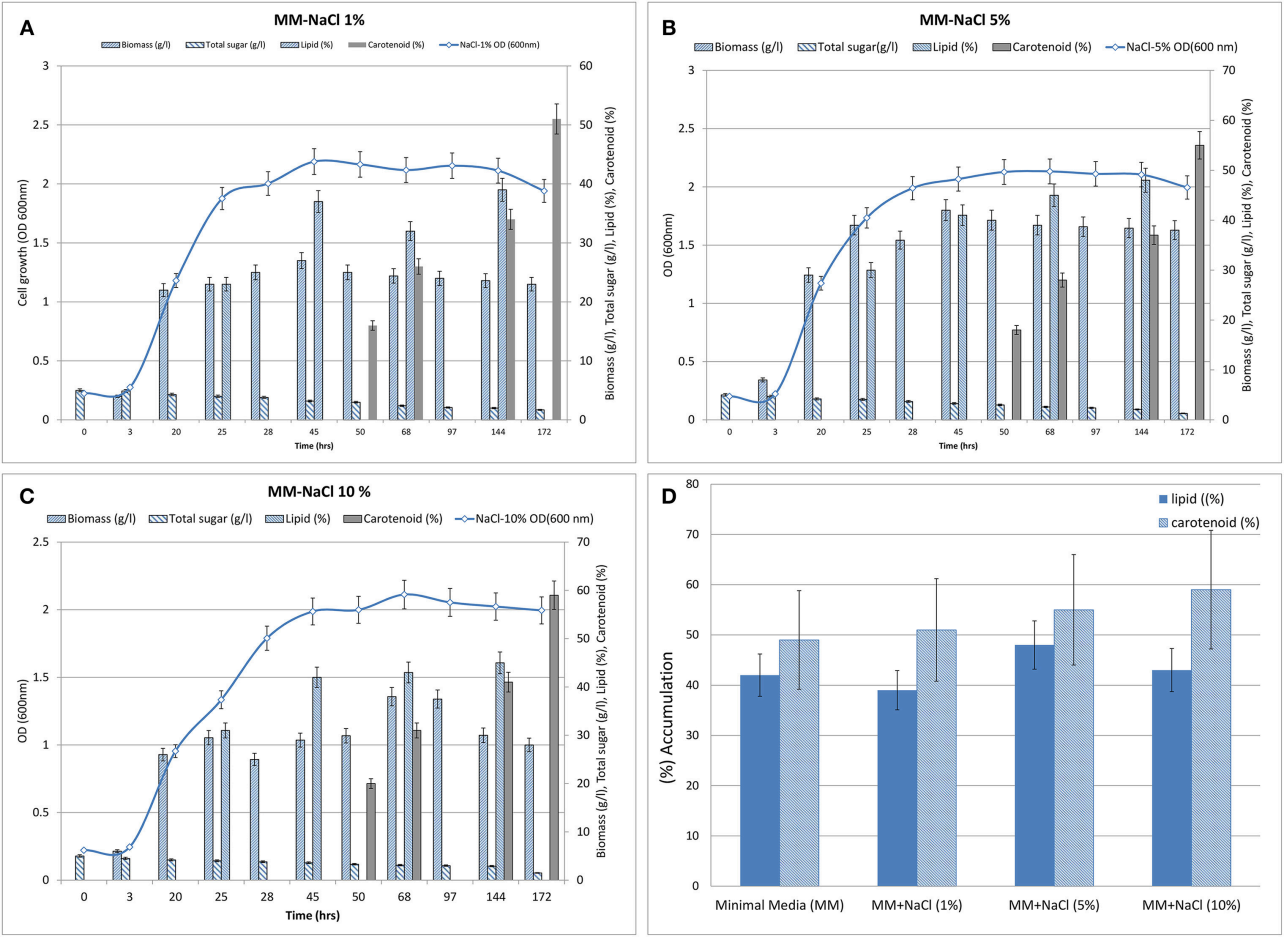
**Biomass (growth), total sugar (g/L), lipid (%, w/w) and carotenoid synthesis (%, w/w) profiles of *R. toruloides* upon treatment. (A)** NaCl, 1% (w/v). **(B)** NaCl, 5% (w/V). **(C)** NaCl, 10% (w/v). **(D)** Graph showing maximum lipid and carotenoid biosynthesis in all three treatments (i.e., with NaCl at 1, 5, and 10% w/v).

To the minimal medium, NaCl was supplemented from 1 to 10% (w/v). When cultivated in minimal medium with sodium chloride (1–10%) in different flasks (Figures 3A-C), the culture displayed a lag phase for up to 3 h from the time of inoculation and then moved in to the log phase. The log phase continued for the next 43 h and subsequently the culture moved on to stationary phase in all the three treatments (i.e., MM containing additional 1, 5, and 10% NaCl). Increasing concentrations of NaCl led to decrease in growth rate and delayed the onset of stationary phase. Glucose remained utilized marginally during the lag phase, but its uptake and assimilation considerably increased after 3 h of culture time as estimated by the amount of residual sugar present in the medium. In the control flask (without additional NaCl), glucose almost exhausted around 172 h of growth while in the flasks with osmotic stress, residual glucose was found to be 1.25–0.4 g after 172 h of culture time.

Lipid extraction was carried out at different time points between 25 and 144 h of growth because fat globules were observed in Sudan Black stained cells around that time (Figure 2). Maximal lipid accumulation of about 39, 48, 43% (w/w) was observed when 1, 5, and 10% additional NaCl was added to MM. Approximately 51, 55, and 59% of carotenoids accumulated after ~170 h in the three treatments (1, 5, 10% NaCl; Figure 3D). The treatment containing 5–10% NaCl therefore showed concomitant production of the metabolites. NaCl was selected as the process variable since it is a potential stress factor that has been reported to have significant effects in cellular metabolism of microbes (Marova et al., 2010, 2012).

### Statistical optimization

A three factor CCD under RSM was employed to estimate the effect of interactions in between the process variables (glucose, NaCl, and culture time) used. The experiments were planned and executed at different variable levels (Table 1) as suggested by the design model. (Table 2) shows the outcomes of each experiment executed as against the predicted values. The responses for growth ranged from 15.56 to 52.32 g in run numbers 11 and 17, respectively. The ratio of the maximum response to the minimum was found to be 3.36. Similarly, the response range for lipid was found to be from 15.59 to 26.49 (run no. 16 and run no. 1 and 4, respectively). Generally, if the response ratio obtained is higher than 10, an exponential or log transformation of data is required. As the ratio obtained here was < 10, analysis was carried out without any power transformation in data obtained. For carotene synthesis, the response ranges were found to be from 1.76 to 36.90 (Run no.10 and run no. 1). As the ratio of maximum to minimum is 28, which is more than 10, a power transformation may be required. The model suggested by curve fitting of responses obtained for growth, fitted the response surface reduced quadratic model with the sequential model sum of squares equal to 1791.00, *F*-value 160.77 and *p*-value of < 0.0001. The model was tested by using ANOVA including Fischer's test and is was found to be significant to explain the responses all over the selected design space. The model had 8 degrees of freedom with 6 degrees of freedom for “Lack of Fit” and 3 degrees of freedom for “Pure Error.” The model was modified to reduced quadratic model after ANOVA (Table 4A).

**Table 4A:** **ANOVA table for growth of *R. toruloides***.

**Source**	**Sum of squares**	***df***	**Mean square**	***F*-value**	***p*-value (Prob > *F*)**	**Significance**
Model	1791.00	8	223.88	160.77	< 0.0001	significant
A-glucose	1382.22	1	1382.22	992.63	< 0.0001	
B-NaCl	43.65	1	43.65	31.35	0.0003	
C-culture time	228.84	1	228.84	164.34	< 0.0001	
AB	7.20	1	7.20	5.17	0.0490	
AC	11.16	1	11.16	8.02	0.0197	
A^2^	14.97	1	14.97	10.75	0.0095	
B^2^	11.55	1	11.55	8.29	0.0182	
C^2^	79.76	1	79.76	57.28	< 0.0001	
Residual	12.53	9	1.39			
Lack of fit	12.53	6	2.09			
Pure error	0.000	3	0.000			
Corr total	1803.53	17				

**Figure 4:**
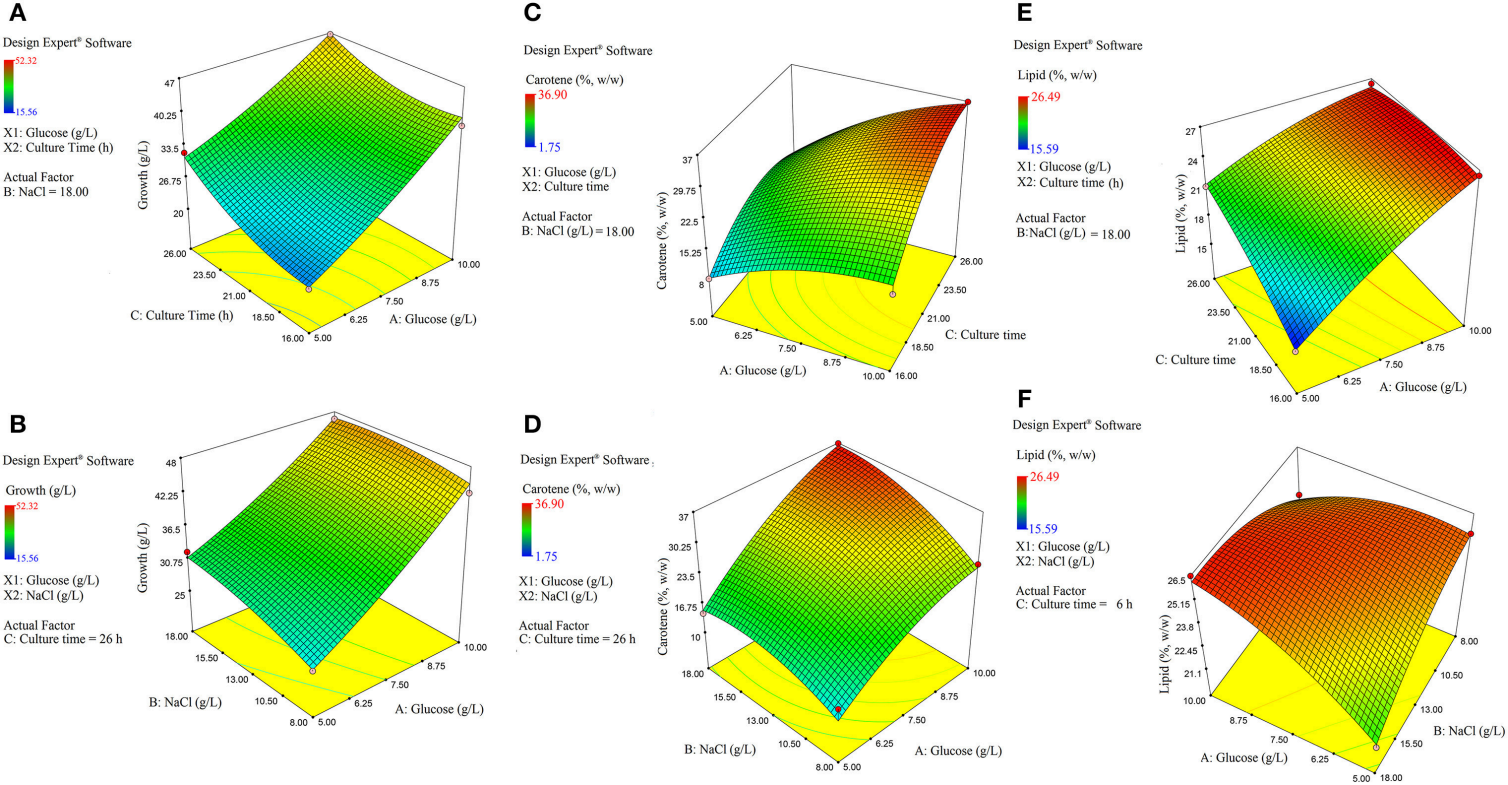
**Response surface plot. (A)** Effect of culture time (h) vs. glucose (g/L) on growth. **(B)** Effect of NaCl (g/L) vs. glucose (g/L) on growth. **(C)** Effect of culture time (h) vs. glucose (g/L) on the carotene synthesis. **(D)** Effect of NaCl (g/L) vs. glucose (g/L) on carotene synthesis. **(E)** Effect of culture time (h) vs. glucose (g/L) on lipid production. **(F)** Effect of NaCl (g/L) vs. glucose (g/L) on lipid production.

**Figure 5:**
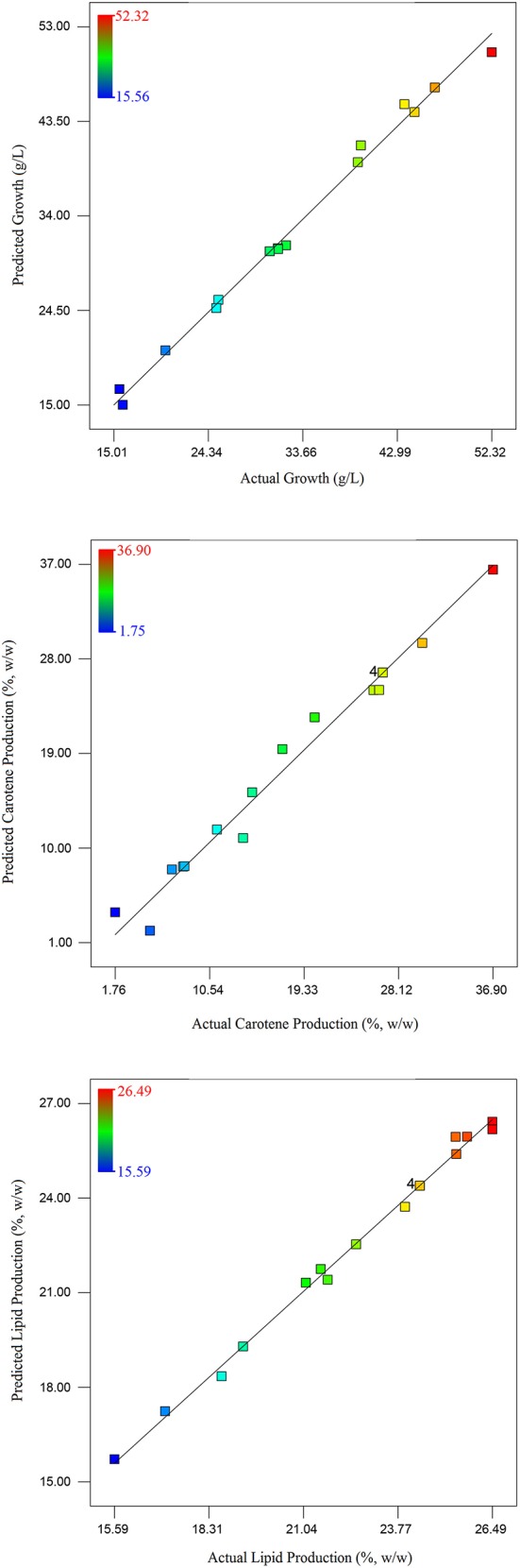
**Parity plot between actual and predicted values of culture growth, carotene synthesis, and lipid production**.

The model interaction terms BC had higher *p*-values (>0.05) and lower *F*-value; therefore, it was considered “not significant” and hence was not taken in to account for model calculations. Therefore, the interaction term was removed from the model for its simplification and to make the curve fitting better. The *F*-value for glucose, NaCl and culture time were found to be highly significant at 992.63, 31.35, and 164.34, respectively. The effect of glucose was much pronounced as compared to NaCl or culture time. Overall, the selected Response surface model had a *F*-value of 160.77, with *p* < 0.0001, showed that the model is valid and there can be only 0.001% chance that this large *F*-value can occur due to noise or outliers. The ANOVA analyses for carotene and lipid production are presented in Tables 4B,C. Analysis of carotene production and lipid synthesis has been performed in similar way. The predicted *R*^2^ (pred. *R*^2^) by ANOVA should be in agreement with adjusted *R*^2^ (adj. *R*^2^), which means that the values should be closer and within the significance range. For growth model, the pred. *R*^2^ and adj. *R*^2^ were 0.9931 and 0.9869, respectively showing a reasonable agreement with each other. Adequate precision quantifies the signal to noise ratio and a value more than 4 is desirable. For growth, this value was measured to be 42.455. (Tables 4A–C) shows a complete analysis of the remaining experimental outcomes on lipid and carotene synthesis in addition to growth and (Table 3) shows the same analysis as a brief summary. The predicted response and the interactions between the participating media components can be depicted pictorially by 2-D contour graphs or by a 3-D plot, also called as response surface plot. (Figures 4A–F) represents the response surface graphs showing interactions and responses over infinitesimally large combinations of two process variables at various levels. (Figure 4A) shows the increase in growth with increase in glucose concentration (from 5 to 10 g/L) as well as culture time (16–26 h), while the variation in NaCl concentration does not play a major role in associated growth (Figure 4B). Carotene production is largely affected by the presence of glucose and culture time (Figure 4C). A higher concentration of glucose increases the amount of carotene synthesized as compared to the same culture time with lower glucose. As we increase the concentration of NaCl (from 8 to 18 g/L) and glucose (5–10 g/L), the yield of carotene increases, with a maximum of 37% (w/w) at the highest design point (Figure 4D). Lipid production showed a direct dependence on culture time and glucose (Figure 5E), on the other hand, interactions between NaCl and glucose affecting lipid production were not so straightforward. Increase in NaCl concentration affects lipid production negatively at lower glucose concentrations (Figure 4F), but as glucose is increased the negative effect of NaCl is compensated for lipid production. Maintaining the initial glucose at a higher concentration makes the lipid production proportional to the amount of NaCl added to the medium. The predicted run was carried out using the optimized conditions as predicted by the RSM model at glucose 10 g/L, NaCl 17.7 g/L and culture time of 24.7 h. The predicted growth, carotene and lipid production were 45.1 g/L, 36.5 and 26.49% (w/w), respectively. The predicted runs were performed in real time in two sets of triplicate runs. The average growth obtained was 44.6 g/L, carotene 36.2% (w/w) and lipid 27.2% (w/w).

**Table 4B:** **ANOVA analysis of carotene production**.

**Source**	**Sum of squares**	***df***	**Mean square**	***F*-value**	***p*-value (Prob > *F*)**	**Significance**
Model	1730.96	8	216.37	57.71	< 0.0001	significant
A-glucose	544.68	1	544.68	145.29	< 0.0001	
B-NaCl	213.06	1	213.06	56.83	< 0.0001	
C-culture time	497.49	1	497.49	132.70	< 0.0001	
AB	25.26	1	25.26	6.740	0.0289	
AC	50.00	1	50.00	13.34	0.0053	
A^2^	96.41	1	96.41	25.71	0.0007	
B^2^	109.00	1	109.00	29.07	0.0004	
C^2^	328.69	1	328.69	87.68	< 0.0001	
Residual	33.73	9	3.74			
Lack Of fit	33.73	6	5.62			
Pure error	0	3	0			
Cor total	1764.70	17				

**Table 4C:** **ANOVA for lipid synthesis**.

**Source**	**Sum of squares**	***df***	**Mean square**	***F*-value**	***p*-value (Prob > *F)***	**Significance**
Model	180.416	9	20.04	195.09	< 0.001	significant
A-glucose	69.652	1	69.65	677.88	< 0.001	
B-NaCl	0.742	1	0.742	7.23	0.027	
C-culture time	53.436	1	53.43	520.06	< 0.001	
AB	21.353	1	21.35	207.81	< 0.001	
AC	17.082	1	17.08	166.24	< 0.001	
BC	3.264	1	3.26	31.76	< 0.000	
A^2^	7.969	1	7.96	77.56	< 0.001	
B^2^	8.005	1	8.00	77.90	< 0.001	
C^2^	4.954	1	4.95	48.21	0.001	
Residual	0.821	8	0.10			
Lack of fit	0.821	5	0.16			
Pure error	0	3	0			
Cor total	181.238	17				

## Discussion

Lipids or fatty acid alkyl/methyl esters synthesized and accumulated by microorganisms (oleaginous microbes) are considered as prudent alternatives to fossil fuels. Long chain fatty acids, are excellent substrates for biodiesel production (Najafi et al., 2007). Research for biofuels has been progressing considerably, with recent advances in 3rd and 4th generation of biofuels. The 3rd generation of biofuels and their production aims at creation of sustainable energy source and reduces the CO_2_ emission, if not zero. Both oleaginous algae and yeast have been employed for the generation of biodiesel as these organisms accumulate significant amounts of lipids, which can be easily converted to biodiesel by various methods of transesterification (Ratledge, 1982). However, algal cell walls and pigments are a challenge for procuring and downstream processing of these accumulated lipids. Yeasts on the other hand, alleviate this limitation due to their cellular structure, and ease of the biodiesel recovery process. Yeasts can be cultivated using various types of substrates considered as waste, leading to effective waste management with an advantage of being coupled to biodiesel production. The fuel resulting from currently synthesized 3rd generation biofuels is indistinguishable from its petroleum counterparts (Zhu et al., 2008; Saenge et al., 2011). This generation of fuels are also called advanced biofuels or green hydrocarbons.

*R. toruloides* is one of the oleaginous microorganisms with the potential to synthesize and accumulate lipids intracellularly up to ~70% of its total biomass under normal stress free culture conditions. The organism can also grow on various carbon sources such as pentoses and glycerols apart from hexoses (glucose). This property has made *R. toruloides* an important oleaginous organism for production of lipids and other industrially important commodities such as carotenoids (composed of β-carotene, astaxanthin, etc). Lipids are produced and accumulated during the log phase of growth when increase in biomass is exponential and utilization of sugar is rapid (rate 0.032 g/Lh^−1^). On the other hand, carotenoids being secondary metabolites, are produced after beginning of the stationary phase and continued until death phase sets in. Carotenoids have recently been gaining importance as natural food colorants, antioxidant properties and their speculated anti-cancer activity (Frengova and Beshkova, 2009; Saenge et al., 2011).

Carbon/nitrogen ratios of the growth medium influence the production of lipids by affecting the activity of AMP dependent isocitrate dehydrogenase in the TCA cycle (Ratledge, 1982), thereby channelizing the acetyl CoA for lipid biosynthesis. A medium with a high C/N ratio tends to produce lipids rather than carotenoids (Somashekar and Joseph, 2000), however, as growth progresses, the C/N ratio changes that leads to the induction of secondary metabolite production, e.g., carotenoids. Therefore, a balanced C and N ratio needs to be explored for lipid production in the initial phases, followed by carotenoid production during later stages of growth. Previous studies also validated our results that increase in carbon content caused increased lipid accumulation and delayed the onset of stationary phase during which carotenoids are produced (Galafassi et al., 2012; Yen and Yang, 2012; Braunwald et al., 2013; Schneider et al., 2013). Bhosale and Gadre (2001) have reported increased production of lipids with increase in carbon (glucose) until about 100 g/l.

Concomitant production of lipids and carotenoids is only possible when media is standardized selectively for the strain under consideration and appropriately optimized for a balanced production of single or multiple metabolites of interest. Saenge et al. (2011) studied the effects of C/N ratio and surfactant concentration on the concomitant production of lipids and carotenoids using RSM on palm oil mill effluent (POME). This observation clarified that there was an interplay of factors other than C and N; mere variations of C and N in synthetic medium may not lead to concomitant lipid and carotenoid production. We intended to delay the onset of stationary phase for maximum biomass generation, thereby increasing the accumulation of lipid (from 42% in control to 48% in treatments) beyond ~25 h of cultivation.

To increase the yield of lipids and pigments, a combined effect of minimal medium supplemented with carbon and nitrogen ratios was proposed along with stress condition. *R. toruloides* CCY 20-2-26 showed altered morphology, growth and production of biomass, carotenoids, and ergosterol under the influence of osmotic (2–10% NaCl) stress, oxidative (2–10 mM H_2_O_2_) stress, and combined stress factors (Marova et al., 2010). Under high stress 2–3 times increase of β-carotene was observed. In this present study, carotenoid accumulation increased from 51 (1% NaCl treatment) to 59% (10% NaCl treatment) suggesting almost 1% increase per 1% additional NaCl added. Stress conditions also improved lipid accumulation by ~8% (in 5% NaCl) as compared to control. After ~144 h of growth lipid and carotenoids were concomitantly produced and therefore the culture was ready to be harvested at this time for downstream processing. Maximum production of carotenoids however, occurred after 170 h due to maximal depletion of carbon and lower C/N ratio at this time. We also inferred that increasing the carbon content of the medium to 10 g/l significantly increased the accumulation of lipids, however, addition of stress factors did not increase the biomass but resulted in concomitant production of lipids and carotenoids with improved productivity (Figures 3A–C; Table 5A), by decreasing the total time of cultivation. Productivity of other oleaginous yeasts in other media (Table 5B) were lower than the values obtained by our studies using NaCl.

**Table 5A:** **Showing yield and productivity of lipids and carotenoids upon addition in control and the three treatments**.

		**Minimal medium (control)**	**MM-1% NaCl**	**MM-5% NaCl**	**MM-10% NaCl**
Biomass (g/L)		31	25	42	38
Lipid	Lipid%	42	39	48	43
	Yield (Y_P/S_)	6.19	8	15	12.7
	Yield (Y_P/x_)	26	26.6	30	28
	Productivity (gL^−1^h^−1^)	0.277	0.270	0.333	0.312
Carotenoid	Carotenoid%	49	51	55	59
	Yield (Y_P/S_)	18.1	23.3	28.4	32.5
	Yield (Y_P/x_)	17	17.5	18.5	20.5
	Productivity (gL^−1^h^−1^)	0.284	0.296	0.319	0.343

**Table 5B:** **Comparative kinetics study of oleaginous yeast strains**.

**Oleaginous yeast strain**	**Biomass (g/l)**	**Growth medium**	**Lipid content**	**Lipid productivity (gL^−1^h^−1^)**	**Carotenoid content (%)**	**Carotenoid productivity gL^−1^h^−1^)**	**References**
*R. toruloides*	42	MM	48%	0.333	55	0.319	This study
*R. toruloides* NCYC 921	69.64	MM	27.57%	0.40	59	0.29	Dias et al., 2015
*Cryptococcus musci* JCM 24512	39	MM	1.49 (g/l)	0.37 (gL^−1^day^−1^)	NA^*^	NA^*^	Tanimura et al., 2014
*Yarrowia lipolytica* JMY4086	60	YPD medium	31%	0.43	NA^*^	NA^*^	Magdalena et al., 2015
*Torulaspora globosa* YU5/2	9.43	Yeast medium	0.2 (g/l)	0.025	NA^*^	NA^*^	Papone et al., 2016

MM, Minimal medium;

* NA, not applicable.

Earlier studies showed that exposition of red yeast cells to all tested stress factors resulted in higher production of carotenoids as well as ergosterol, while biomass production showed only minor changes (Marova et al., 2010). Similarly, in our study *R. toruloides* produced ~23% more lipids and ~20% more carotenoids after ~144 h of growth as compared to control, however, the OD (600 nm) at this point of time remained almost the same in control and the three treatments.

Recently, production of lipids by the yeast *R. glutinis* on different carbon sources (dextrose, xylose, glycerol, mixtures of dextrose and xylose, xylose and glycerol, and dextrose and glycerol) was explored (Easterling et al., 2009). In earlier studies, the highest lipid production of 34% TAG on a dry weight basis was measured with a mixture of dextrose and glycerol as carbon source, while in this study the highest amount of lipid production was 48% (dry cell weight/vol) under stress conditions having MM with 5% NaCl. To increase the yield of these pigments along with improved biomass production, combined effects of medium with variable carbon and nitrogen sources and salt stress was tried. The production of lipid and carotene-enriched biomass was carried out in flasks as well as in lab-scale fermenter. The highest concomitant production of biomass was obtained under salt stress in minimal medium treated with 5% NaCl.

Taking inputs from these experiments, statistical modeling and optimization of biodiesel and carotene production was performed. Statistical methods are preferable as these account for the interaction of different media and other physico-chemical conditions. The interaction in between medium components affect cell growth and lipid production by influencing the metabolism of the culture. This may result in absolute or differential switching on/off of metabolic pathways (Ageitos et al., 2011). In the statistical experiments, highest growth was observed in run no. 17 that included high concentration of glucose with 13 g/L glucose. As growth depends on the amount of carbon present to be converted into biomass, requirement of glucose was anticipated. The carotenoid production maximized with increase in salinity induced by adding large amounts of NaCl (18 g/L). The response surface model selected on the basis of ANOVA that best fitted the model equation was reduced quadratic model for growth and carotenoid production while the model for lipid production was a quadratic model. The analysis of all the three models have been presented in Tables 3, 4A–C. Response surface analysis revealed fairly good interactions between glucose (A) and NaCl (B) with *p* < 0.05 for growth and carotene and < 0.001 for lipid production. The *F*-value for lipid production was highest among all the models that suggests that the media components interacted significantly, and had a profound effect on its production when compared to growth or carotene production. Higher *F*-value shows better fitting of the data in the statistical model and therefore, the reliability of predicted conditions also increases. NaCl is said to induce osmotic stress that results into switching of metabolism in *R. toruloides* and force cellular machinery toward lipid synthesis and accumulation. Numerical optimization with an aim to maximize lipid and carotenoid production, without perturbing the cell growth range for the model resulted into optimized conditions at 10 g/L glucose (A), 17.78 g/L NaCl (B) with a culture time of 24.7 h. In all the three cases, the higher *F*-values indicated that there was only 0.01% chance that this large *F*-value can occur due to noise. The coefficient of determination in all the three cases were found to be >0.98 (Table 3) which indicated a perfect fit of the predicted responses compared to the obtained responses in real time. (Table 2) lists down the obtained vs. predicted values of responses and (Figure 5) shows the parity plot, indicating an aptness and accuracy of the developed RSM model. The obtained results indicated the usability and aptness of the selected RSM model to study the interactions of glucose and NaCl on the concomitant production of carotene and lipids under osmotic stress conditions in *R. toruloides.*

We studied a range of NaCl concentrations to check its effect on concomitant production of lipids as well as carotenoids through statistical modeling. *R. toruloides* can be a potent alternative for sustainable biofuel production as opposed to other biomass due to its flexible growth characteristics and ability to utilize various media modified with stress factor(s), for higher productivity and yield as shown by our experiments. The addition of osmotic stress factors would also decrease the chances of contamination by other microbes during fermentation in larger volumes. In this study, we achieved 36.2% (w/v) and 27.2% (w/v) production of carotene and lipids, respectively, under osmotic stress with high salt concentrations.

## Author contributions

GS conducted experiments, AJ planned experiments. SH and KB oversaw the work. DP wrote and reviewed the article and instructed GS and planned the experiments with AJ. AK was Co-PI in project that funded this work; not contributed to manuscript.

### Conflict of interest statement

The authors declare that the research was conducted in the absence of any commercial or financial relationships that could be construed as a potential conflict of interest.

## Supplementary material

Supplementary Figure 1 **Lipid and carotenoid production and growth profile of *R. toruloides* in the presence of 0.5, 5, and 10 g/L glucose in MM**.
