# Supplementary figures and images for: Concomitant Production of Lipids and Carotenoids in Rhodosporidium toruloides under Osmotic Stress Using Response Surface Methodology
Source: Front Microbiol. 2016 Oct 25;7:1686. doi: 10.3389/fmicb.2016.01686 (PMC5078724; doi:10.3389/fmicb.2016.01686)

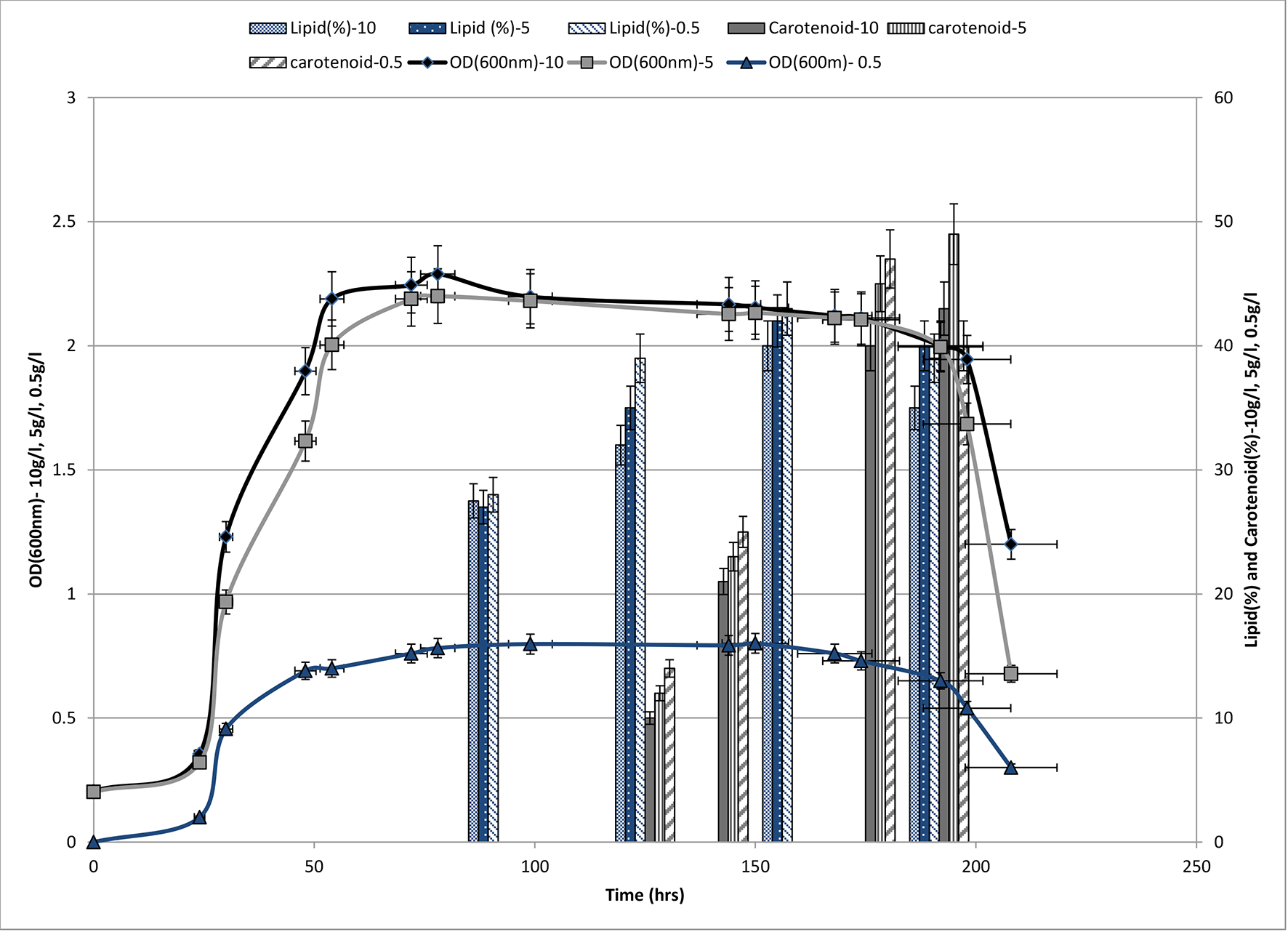

Supplement: Supplementary Figure 1 — Lipid and carotenoid production and growth profile of R. toruloides in the presence of 0.5, 5, and 10 g/L glucose in MM. [file Image1.TIF]
